# A New Variant among Newcastle Disease Viruses Isolated in the Democratic Republic of the Congo in 2018 and 2019

**DOI:** 10.3390/v13020151

**Published:** 2021-01-20

**Authors:** Augustin T. Twabela, Lam Thanh Nguyen, Justin Masumu, Patrick Mpoyo, Serge Mpiana, Julienne Sumbu, Masatoshi Okamatsu, Keita Matsuno, Norikazu Isoda, Bianca Zecchin, Isabella Monne, Yoshihiro Sakoda

**Affiliations:** 1 Laboratory of Microbiology, Department of Disease Control, Faculty of Veterinary Medicine, Hokkaido University, Sapporo, Hokkaido 060-0818, Japan; ttaugushahu@vetmed.hokudai.ac.jp (A.T.T.); ntlam@ctu.edu.vn (L.T.N.); okamatsu@vetmed.hokudai.ac.jp (M.O.); matsuk@czc.hokudai.ac.jp (K.M.); nisoda@vetmed.hokudai.ac.jp (N.I.); 2 Central Veterinary Laboratory of Kinshasa, Kinshasa I/Gombe 012, Democratic Republic of the Congo; jmasumu@hotmail.com (J.M.); carimemarcia@gmail.com (P.M.); drsmpiana@yahoo.fr (S.M.); julienne_s2002@yahoo.fr (J.S.); 3 Department of Veterinary Medicine, Campus II, College of Agriculture and Applied Biology, Can Tho University, 3/2 Street, Ninh Kieu, Can Tho 900000, Vietnam; 4 Unit of Risk Analysis and Management, Hokkaido University Research Center for Zoonosis Control, Sapporo, Hokkaido 001-0020, Japan; 5 Global Station for Zoonosis Control, Global Institution for Collaborative Research and Education (GI-CoRE), Hokkaido University, Sapporo, Hokkaido 060-0818, Japan; 6 Istituto Zooprofilattico Sperimentale delle Venezie, 35020 Legnaro (Padova), Italy; bzecchin@izsvenezie.it (B.Z.); imonne@izsvenezie.it (I.M.)

**Keywords:** new variant, Newcastle disease virus, chicken outbreak, Democratic Republic of the Congo

## Abstract

Newcastle disease (ND) is a highly transmissible and devastating disease that affects poultry and wild birds worldwide. Comprehensive knowledge regarding the characteristics and epidemiological factors of the ND virus (NDV) is critical for the control and prevention of ND. Effective vaccinations can prevent and control the spread of the NDV in poultry populations. For decades, the Democratic Republic of the Congo (DRC) has reported the impacts of ND on commercial and traditional poultry farming systems. The reports were preliminary clinical observations, and few cases were confirmed in the laboratory. However, data on the phylogenetic, genetic, and virological characteristics of NDVs circulating in the DRC are not available. In this study, the whole-genome sequences of three NDV isolates obtained using the next-generation sequencing method revealed two isolates that were a new variant of NDV, and one isolate that was clustered in the subgenotype VII.2. All DRC isolates were velogenic and were antigenically closely related to the vaccine strains. Our findings reveal that despite the circulation of the new variant, ND can be controlled in the DRC using the current vaccine. However, epidemiological studies should be conducted to elucidate the endemicity of the disease so that better control strategies can be implemented.

## 1. Introduction

Newcastle disease (ND), which is caused by the Newcastle disease virus (NDV), is a highly contagious and devastating infectious disease that affects poultry and wild birds worldwide. The NDV is also known as the avian paramyxovirus type 1 (APMV-1) and has been classified in the family *Paramyxoviridae*, subfamily *Avulavirinae*, and genus *Metaavulavirus* [1]. The NDV has a negative-sense RNA genome comprising 15,186–15,198 nucleotides for the whole genome and encodes for six proteins in the following order: nucleoprotein (NP), phosphoprotein (P), matrix (M), fusion (F), hemagglutinin-neuraminidase (HN), and large polymerase (L) [2,3]. In addition, two nonstructural proteins, V and W, are generated during P gene translation through RNA editing [4]. The HN and F proteins are surface glycoproteins that play an important role in the infection, pathogenicity, and antigenicity of NDV [5,6]. Based on the genome size and nucleotide sequences of the F and L genes, NDV strains have been divided into class I and class II [7]. Class II NDVs have higher genetic diversity, including 20 newly identified genotypes named I to XXI (excluding XV, because it contains recombinant viruses), which comprise virulent and avirulent strains [8].

More than 250 avian species have been identified to be susceptible to NDV with varying pathogenicity [9]. However, poultry populations possess high susceptibility to NDV, and the pathogenicity varies by strain. Strains are classified based on their pathogenicity, as highly virulent (velogenic strain), moderately virulent (mesogenic strain), and avirulent (lentogenic strain) [10]. The virulence of NDV is primarily attributed to the function of the F gene [11]; however, other genes modulate the capacity of virus replication which can lead to virulence [12]. The characteristics and epidemiological data on NDVs circulating in poultry and wild bird populations within a specific region provide useful information for ND control and prevention.

In the Democratic Republic of the Congo (DRC), the poultry industry impacts the economy and public health through food safety. Production is mostly based on commercial and modern chicken farming systems with broilers and layers; however, small-scale and backyard farming systems are employed in rural areas. Owing to its high commodity value, poultry production has a vital role in the economy.

During the last two decades, outbreaks and resulting mortality attributed to NDV infection in poultry have been frequently reported across various farming systems in the DRC. Most case reports were based on clinical manifestations, whereas few laboratory-confirmed cases were notified [13]. No investigations were performed to confirm or characterize the reported cases; therefore, there is lack of epidemiological and genetic profiling of NDV strains circulating in poultry populations of the DRC. This study aimed to characterize NDVs isolated in the DRC during the period 2018–2019. The phylogenetic, pathogenic, and antigenic traits of NDVs were evaluated to elucidate the features of newly isolated strains and to provide a basis for the development of mitigation measures for the control and prevention of ND throughout the DRC.

## 2. Materials and Methods

### 2.1. Sample Collection 

Tracheal and cloacal swab samples were collected from diseased chickens during two different outbreaks that occurred in 2018 and 2019 in the Tshuapa and Kinshasa provinces of the DRC, respectively (Table 1; Figure S1). All chickens were raised in traditional farming systems without any vaccination history. The collected swabs were put into a viral transport medium (Teknova, 2290 Bert Dr, Hollister, CA 95023, USA), and aliquots were shipped on dry ice to Hokkaido University, Sapporo, Japan, for the diagnosis of avian influenza and ND.

### 2.2. Virus Isolation and Identification

The swab supernatant was inoculated in 10-day-old embryonated chicken eggs obtained from a conventional chicken flock that tested negative for the NDV antibody via a hemagglutination inhibition (HI) test [14] using the antigen NDV/duck/Hokkaido/1/1977 strain available in our laboratory. After 72 h of incubation at 37 °C, the presence of NDV was confirmed in the allantoic fluid by a hemagglutination (HA) test. All HA-positive samples were then subjected to the HI test against a hyperimmune antiserum anti-NDV from our laboratory library according to the World Organization for Animal Health (OIE) guidelines [14].

### 2.3. Sequencing, Phylogenetic, and Genetic Analyses

Viral RNA was extracted from the infectious allantoic fluid using TRIzol LS reagent (Life Technologies, Carlsbad, CA, USA) according to the manufacturer’s instructions. The whole-genome sequence was obtained using next-generation sequencing. Briefly, the MiSeq libraries were prepared using the NEBNext Ultra RNA Library Prep Kit for Illumina (New England Biolabs, Ipswich, MA, USA) and were sequenced using the MiSeq system (Illumina, San Diego, CA, USA). The sequence reads were mapped to the reference sequence of NDV. The consensus sequence was reconstructed until all mismatches were solved using the CLC Genomic Workbench, version 12.0 (CLC bio, Aarhus, Denmark).

For phylogenetic analysis, two different datasets were prepared. One contained the pilot dataset of the complete NDV F gene sequence proposed by Dimitry et al. [8], in which three sequences of the Congo isolates were inserted. The other dataset contained the whole-genome sequence of the representative NDV downloaded from GenBank (*n* = 68), which belonged to each of the 20 genotypes, and three full genome sequences of the Congo isolates were inserted. The two datasets were aligned in BioEdit version 7.0.5.3, and the maximum-likelihood method using the general time-reversible model with gamma distribution was run in MEGA7 v 7.0.23 [15] to construct the phylogenetic tree.

The identities of the Congo and other NDV isolates were assessed using the basic local alignment search tool (BLAST); and changes in the amino acid (AA) position of the F protein were assessed using GENETYX v.15.0.1 (GENETYX Corp. Tokyo, Japan). The evolutionary divergence among sequence pairs of Congo NDVs and other viruses of different genotypes in class II was calculated in MEGA 7 [15].

### 2.4. Pathogenicity Assessment of NDV Isolates

The pathogenicity of the two Congo isolates (NDV/Ck/DRC/PL33/2018 and NDV/Ck/DRC/PL219/2019) was determined based on the Intracerebral Pathogenicity Index (ICPI) and mean death time (MDT) assays, as recommended by the OIE [14]. Briefly, for the ICPI assessment, 0.05 mL fresh infectious allantoic fluid was diluted 10-fold with sterile phosphate-buffered saline (PBS) and then intracerebrally inoculated in 10 24-h-old chicks hatched in our laboratory; the eggs were obtained from an NDV-unvaccinated flock. The inoculated chicks were monitored for clinical signs and mortality every 24 h for 8 days and were scored as 0 for normal, 1 for sick, and 2 for dead. For the MDT, 0.1 mL of 10-fold diluted allantoic fluid (10^−5^ to 10^−10^) was inoculated in 10-day-old embryonated chicken eggs from the same NDV-unvaccinated flock. The eggs were incubated for 120 h at 37 °C and were candled every 8 h for embryo viability. The number of dead embryos was recorded to calculate the mean lethal dose (MLD) that can kill all inoculated embryos. The MDT was obtained as the mean time of survival after inoculation of the virus with the MLD titer.

### 2.5. Antigenic Characterization

Hyperimmune antisera against two Congo field isolates (NDV/Ck/DRC/PL33/2018 and NDV/Ck/DRC/PL219/2019) and two vaccine strains (I-2 and Hitchner B1) were prepared in 4-week-old chickens immunized thrice with 0.1% formalin-inactivated allantoic fluid, as previously described [16]. A cross-hemagglutination inhibition (cross-HI) test was performed against the homologous and heterologous antigen in a 96-well microtitration plate using 1% chicken red blood cells [14]. Additionally, the serum neutralizing test (SNT) was performed as previously described [17]. Briefly, the prepared antisera were inactivated at 56 °C for 30 min and diluted 10-fold in serum-free minimum Eagle’s medium (MEM, Nissui Pharmaceutical, Tokyo, Japan) containing 0.3 mg/mL L-glutamine (Wako Chemicals, Tokyo, Japan), 100 U/mL penicillin G (Meiji Seika Pharma, Tokyo, Japan), 0.1 mg/mL streptomycin (Meiji Seika Pharma, Tokyo, Japan), and 8 µg/mL gentamicin (TAKATA Pharmaceutical Co., Ltd. Saitama, Japan). The antigen was also diluted in serum-free MEM to obtain the 10^2^ 50% egg infectious dose (EID_50_). The diluted antisera were mixed at equal volumes with a homologous or heterologous antigen, and the mixture was incubated for 1 h at 37 °C. Then, 100 µL of the mixture was added in the confluent monolayer of the chicken embryo fibroblast cells that had been prepared 3 days before. After 1 h of incubation, cells were washed three times with PBS, and then MEM with serum was added. The plate was incubated for 6 days at 37 °C in 5% CO_2_. Then, the cytopathogenic effect (CPE) was observed in each well, and the neutralizing titer was calculated using the Reed and Muench method [18]. The antigenic relatedness was determined by the coefficient of antigenic similarity (R) between each pair of strain according to the method of Archetti and Horsfall [19], where an R-value of 0.67 < R < 1.5 indicated no significant antigenic difference; 0.5 < R < 0.67 indicated a minor difference; and R < 0.5 indicated a major difference between the two virus strains.

### 2.6. Vaccine Potency Test

To evaluate the potency of an ND vaccine used locally in the DRC to protect poultry from newly isolated viruses, 4-week-old chickens (hybrid-breed Julia and Boris brown, which were hatched and reared in our laboratory and tested free of the NDV antibody) were categorized into three groups of 10 birds each. Two groups were assigned for vaccination, and the chickens were inoculated with either the I-2 or Hitchner B1 (B1) vaccine by the eye drop method. The I-2 vaccine strain, which is used in the DRC for small-scale chicken farms [20], was prepared according to the user manual [21]. Briefly, the I-2 vaccine strain master seed that was shipped with the swab samples from DRC was inoculated into 10-day-old embryonated chicken eggs from an unvaccinated flock and tested negative for the NDV antibody. After 3 days of incubation at 37 °C, the allantoic fluid was collected and clarified by centrifugation for 5 min at 2500 rpm. The allantoic fluid containing 10^9^ EID_50_/mL titer was mixed at equal volumes with a sterile stabilizer of 2% gelatin (Wako Chemicals, Tokyo, Japan) in distillated water. For the vaccination, 30 µL of the prepared vaccine was inoculated by the eye drop method. The B1 was purchased in Japan (Nisseiken Co., Ltd. Tokyo, Japan) and was used following the manufacturer’s instructions. The remaining group of chickens was assigned to the control group that received no vaccination. At 7 days after the first vaccination, a boost vaccination was performed by eye drop; 15 days after the first shot and when the antibody titer reached 128 HI or more against each vaccine strain according to HI assay [14], 100 µL 10^2^ EID_50_ of infectious allantoic fluid of the NDV/Ck/DRC/PL219/2019 isolate was intranasally inoculated in the chickens of all three groups. The chickens were monitored daily for 14 days to record the survival and clinical signs. Tracheal and cloacal swab samples were collected at 0-, 3-, 5-, 7-, and 14-days post-infection (dpi) for virus shedding assessment. All swabs were placed separately into 2 mL of the viral transport medium. After vortex and clarification by centrifugation for 5 min at 2000 rpm, the supernatant of each swab was inoculated into four 10-day-old chicken embryonated eggs that were obtained from an unvaccinated flock. After 3 days of incubation at 37 °C, the infectivity of each swab was confirmed by an HA test of the inoculated allantoic fluid.

### 2.7. Ethical Statement

For sample shipment, approval was obtained from the Central Veterinary Laboratory of Kinshasa, DRC, number: 534/ADM/LVC/SA/YB/148/2018 of June 13, 2018.

The animal experiments were conducted in the animal biosafety level 3 facility of the Faculty of Veterinary Medicine, Hokkaido University, Sapporo, Japan; which has been certified by the Association for Assessment and Accreditation of Laboratory Animal Care International (AAALAC International) since 2007. The approval number 18-0035 and 18-0037 were obtained for antiserum preparation and challenge experiments, respectively.

## 3. Results

### 3.1. Virus Isolation and Identification

All three HA-positive samples were identified as NDV by HI test using antiserum prepared against NDV/duck/Hokkaido/1/1977, which was available in the serum library of our laboratory. Among these viruses, two were isolated in the Tshuapa province and one was isolated in the Kinshasa province (Table 1, Figure S1). All samples were collected from sick chickens in the traditional farming system. The nucleotide sequence for the whole genome of the DRC isolates was submitted to the GenBank database under the accession numbers provided in Table 1. 

### 3.2. Phylogenetic and Genetic Analyses

**Figure 1:**
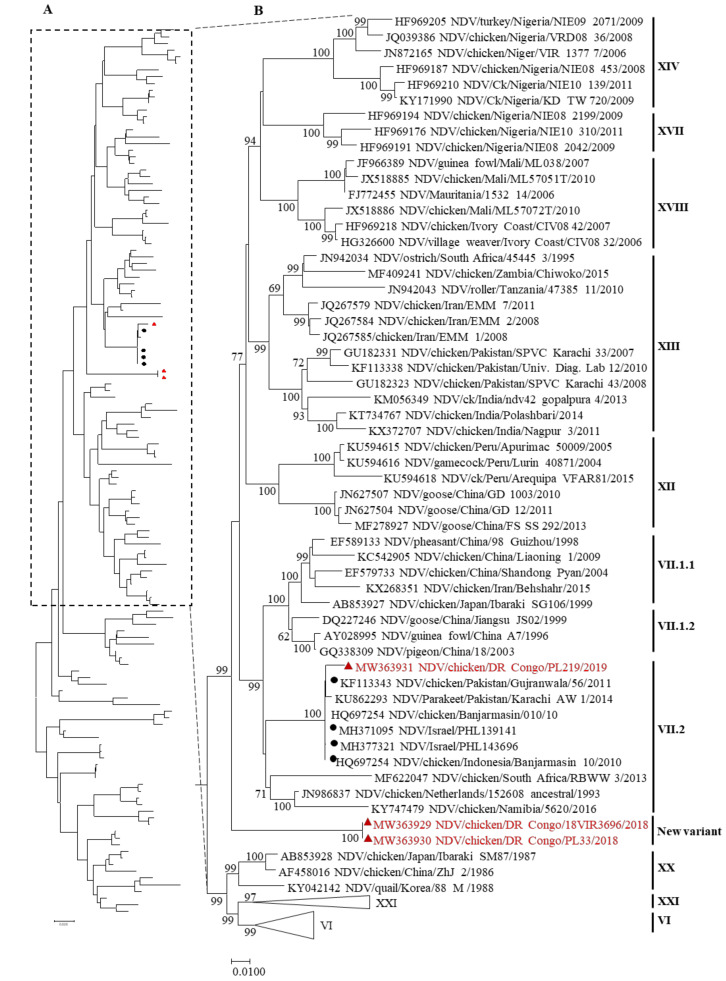
Phylogenetic tree of class II NDV based on the full-length nucleotide sequence of the fusion gene containing 133 sequences including the Congo strains. The analysis was performed in MEGA7 v 7.0.23 using the maximum-likelihood method based on the general time reversible model with 1000 bootstrap replicates. (**A**) The entire phylogenetic tree in which the taxa tips are not shown. (**B**) Part of the tree is shown with all taxa tips indicated; the vertical bar indicates the genotype or subgenotype of each strain group or individual. The triangle and red color indicate the Democratic Republic of the Congo isolates. The black circle indicates the strain in the top five hits of the BLAST search result of the Congo strain NDV/Ck/DR Congo/PL219/2019.

Phylogenic analysis based on the complete F gene sequence revealed that the virus NDV/Ck/DRC/PL219/2019 was clustered in genotype VII with other Asian, some African, and European strains; however, a specific clustering of only Asian/Middle East strains in subgenotype VII.2 was observed. The two viruses NDV/Ck/DRC/18VIR3696/2018 and NDV/Ck/DRC/PL33/2018 which were isolated in the Tshuapa province in 2018 were clustered independently from the other NDV strains in the public database, thus forming a divergent branch in the phylogenetic tree (Figure 1). The same clustering for the F gene was observed in the phylogenetic tree that had been constructed using the whole-genome sequence (Figure S2).

The similarities of the complete F gene sequence between the Congo isolates and other strains were assessed using BLAST search. For the NDV/Ck/DCR/18VIR3696/2018 and NDV/Ck/DRC/PL33/2018 strains, the identity with the ten top-hit strains was 90.53–88.85%, and mostly contained strains from India and Indonesia. For the isolate NDV/Ck/DRC/PL219/2019, the identity was 98–96%, with the top-hit strains coming from Pakistan and Israel (data not shown). To confirm the divergence that was observed in the phylogenetic tree, an evolutionary distance analysis of the three Congo isolates was performed with other NDVs of different genotypes. The result revealed that the genetic distance between the two strains NDV/Ck/DRC/18VIR3696/2018 and NDV/Ck/DRC/PL33/2018 and that of other viruses from all 20 genotypes was >10% (Table 2), which indicated a genetic divergence that has been assigned as a new variant of class II NDVs.

### 3.3. Pathogenicity of the DRC Isolates

Molecular assessment of the cleavage site of the F protein revealed that all three Congo isolates possessed the AA combination motif ^112^RRQKR|F^117^ of the velogenic strains of NDV. In addition, the ICPI values of NDV/Ck/DRC/PL33/2018 and NDV/Ck/DRC/PL219/2019 were 1.84 and 1.95, respectively. The MDT values of NDV/Ck/DRC/PL33/2018 and NDV/Ck/DRC/PL219/2019 were 54.4 and 57.2 h, respectively (Table 1). Considered together, these results indicate the velogenic pathotype of the Congo strains.

### 3.4. Antigenic Characterization

To evaluate the vaccine’s efficacy, it is necessary to determine the antigenic relatedness between the field isolates and vaccine strain. For these newly isolated strains, we sought to compare the antigenicity between field isolates and vaccine strains frequently used in the DRC. The cross-HI test revealed no antigenic difference between the field and vaccine strains, given that the HI titers differed only 2–4-fold. R values of >0.7 obtained from the SNT also showed that there was no antigenic difference between the viruses (Table 3). These results indicated that the newly isolated NDVs in the DRC are antigenically closely related to the vaccine strains used in DRC that were evaluated in this study. 

### 3.5. Vaccine Potency against the Field Isolate

**Figure 2:**
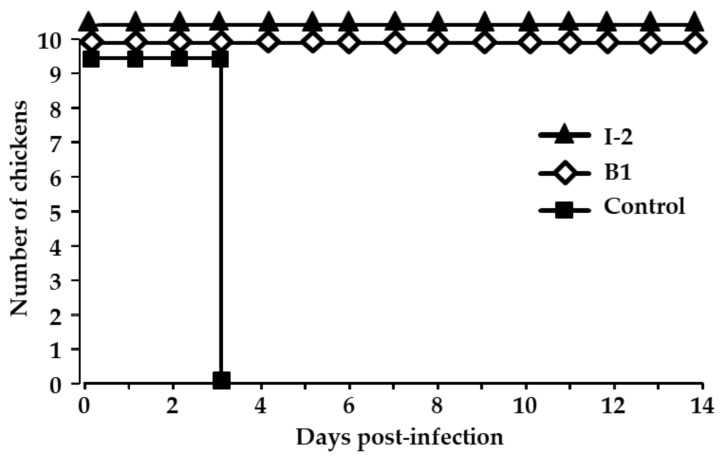
The survival of chickens infected with the Congo NDV field strain NDV/Ck/DRC/PL219/2019. In two groups of 10 birds each, chickens were vaccinated twice by eye drop at 7 day intervals with either I-2 or B1 vaccine. In another group, 10 unvaccinated chickens were assigned as the control. Fifteen days post-vaccination, all chickens were infected intranasally with 10^6^ EID_50_/100 mL of infectious allantoic fluid and were monitored for 14 days.

To ensure the antigenic relatedness between Congo field isolates and the vaccine strains, the animal experiment was conducted by vaccinating and subsequently infecting naïve chickens with the NDV/Ck/DRC/PL219/2019 strain. All the vaccinated chickens in the I-2 and B1 groups survived the infection, and no clinical signs were observed during the 14-day period. However, in the control group, all chickens died at 3 dpi (Figure 2). To assess the virus shedding from surviving chickens, swab samples collected at 3, 5, 7, and 14 dpi were inoculated in eggs. No virus was detected in any of swabs from the chickens in the vaccinated groups (data not shown). This result indicates that the vaccines efficiently protected the chickens from infection and suppressed virus shedding from infected birds.

## 4. Discussion

In the DRC, ND has been reported for a long time in commercial and traditional poultry farms. The reports were mainly clinically based observations that were made during the outbreaks, and few laboratory-based cases were notified [13]. Thus, there is no information on the genetic profile or virological characteristics of circulating NDV strains. In this study, detailed features of three NDV isolates from two different outbreaks were obtained. Phylogenetic analysis of the complete F gene sequence based on a classification by Dimitrov et al. [8] revealed that the viruses NDV/Ck/DRC/18RVI3696/2018 and NDV/Ck/DRC/PL33/2018 isolated in the Tshuapa province were highly divergent from the NDV/Ck/DRC/PL219/2019 virus isolated in Kinshasa belonging to genotype VII. The isolates from the Tshuapa province genetically diverged from all other NDV sequences that are currently available in the referenced public databases.

The topology and the length of the branches of the phylogenetic tree and the genetic distance between these two isolates from Tshuapa province as well as the complete dataset of sequences from all existing genotypes indicates that these isolates may represent a new variant of the NDV in class II. However, we failed to assign these viruses to a new genotype, given their isolation from a single outbreak, because isolation should occur from multiple independent outbreaks, as recommended in the new classification criteria proposed by Dimitrov et al. [8]. The high divergence of these viruses could be owing to virus evolution in naïve poultry populations, which has led to several mutations during the virus replication rather than owing to vaccine immune pressure, which has been reported to increase the occurrence of mutations in the NDV genome [22]. In the countryside of the Tshuapa province, vaccination is rarely administered in indigenous poultry, excluding the possibility of vaccine pressure. We also hypothesize that this NDV variant is a native strain that has never been identified before to be compared with other NDVs circulating in the region. Moreover, the isolate NDV/Ck/DRC/PL219/2019 clustered with other African NDVs in the large genotype VII, but not with NDVs from neighboring countries. However, this virus was closely related to Indonesian and Pakistani strains that clustered together in subgenotype VII.2. We assume that this virus has been introduced from Asia and/or the Middle East, considering their close relatedness. This situation can be explained by the fact that in the DRC, especially in the Kinshasa province where this virus was isolated, the poultry industry and related businesses are conducted mainly by Indian, Pakistani, and Lebanese communities, which may have introduced the virus via live chickens or contaminated materials. A BLAST search of the NDV/Ck/DRC/PL219/2019 isolate produced a top-10 hit among viruses from Pakistan and Israel, which supports the assumption that this virus was introduced from Asia, based on its sequence identity. However, we cannot deny that the lack of genetic data for the NDVs circulating in neighboring countries [23] makes it difficult to identify more closely related viruses in the region. However, considering the transboundary character of NDV, these velogenic isolates from the DRC can spread across the region given the porosity and the poor control measures between country’s borders. 

This study demonstrated that two variants of NDV isolated in the DRC are antigenically closely related to the I-2 vaccine strain that is mostly used in the traditional farming system of the DRC [20], and to the B1 strain that is widely used against ND. Furthermore, the I-2 and B1 vaccine strains were evaluated for their potential ability to protect chickens from newly isolated NDVs in the DRC. The two vaccine strains efficiently protected chickens against lethal infection and suppressed virus shedding from vaccinated birds. Cumulatively, these findings indicate that despite the new variants of NDV detected in the DRC, ND can still be well-controlled by an effective vaccination program that utilizes the currently available vaccines. However, although an effective vaccine is available, the ND is still endemic mainly in the traditional poultry farming systems probably due to the lack of adequate veterinary services in rural zones making it difficult for farmers to access the vaccine [20].This study demonstrates that it is necessary to conduct epidemiological studies to elucidate the endemicity of ND in the DRC, so that the appropriate strategies can be applied for the control and prevention of the disease throughout the DRC.

## Figures and Tables

**Table 1:** Characteristics of the Newcastle disease viruses isolated in the Democratic Republic of the Congo in 2018 and 2019.

Isolate	Date of Isolation	Host	Location	ICPI	MDT	Cleavage Site Amino Acid Motif	Accession Number
NDV/Ck/DRC/18VIR3696/2018	May 2018	Chicken	0.2843 S 20.8851 E	nd	nd	^112^RRQKR|F^117^	MW363929
NDV/Ck/DRC/PL33/2018	May 2018	Chicken	0.2843 S 20.8851 E	1.84	54.4	^112^RRQKR|F^117^	MW363930
NDV/Ck/DRC/PL219/2019	July 2019	Chicken	−4.2436 S 15.5362 E	1.92	57.2	^112^RRQKR|F^117^	MW363931

ICPI: intracerebral pathogenicity index; MDT: mean death time; nd: not determined.

**Table 2:** Estimates of evolutionary divergence over sequence pairs among genotypes of class II NDVs.

Genotype								Number of Base Substitutions Per Site												
	DRC	I	II	III	IV	V	VI	VII	VIII	IX	X	XI	XII	XIII	XIV	XVI	XVII	XVIII	XIX	XX
DRC (*n* = 2)																				
I (*n* = 12)	20.09																			
II (*n* = 3)	21.67	14.20																		
III (*n* = 3)	19.30	12.79	14.66																	
IV (*n* = 2)	15.84	13.02	14.81	10.09																
V (*n* = 6)	15.72	18.56	19.98	16.33	14.24															
VI (*n* = 21)	15.10	19.85	21.10	18.72	16.03	15.25														
VII (*n* = 14)	13.74	19.11	21.75	17.61	14.95	15.54	13.89													
VIII (*n* = 3)	14.46	15.19	1.709	13.63	10.89	11.39	12.09	12.72												
IX (*n* = 3)	19.22	12.16	13.12	9.36	9.53	15.81	18.04	17.22	12.72											
X (*n* = 4)	22.56	12.55	12.07	14.11	14.07	19.60	20.95	20.27	16.54	12.81										
XI (*n* = 3)	25.94	2153	23.24	19.43	14.76	21.38	24.24	24.69	19.74	17.62	22.44									
XII (*n* = 6)	14.89	20.33	23.40	19.15	16.58	15.56	13.77	12.45	13.36	18.71	21.37	25.65								
XIII (*n* = 12)	13.20	19.61	22.14	18.96	15.49	15.37	14.57	11.76	13.02	17.41	20.78	24.42	11.89							
XIV (*n* = 6)	16.49	23.57	26.30	22.92	19.51	18.32	17.13	15.01	15.93	22.49	23.98	29.69	14.50	14.05						
XVI (*n* = 3)	17.64	17.53	19.81	16.35	13.38	14.83	16.13	16.57	11.74	15.62	18.32	22.85	16.97	16.47	19.85					
XVII (*n* = 3)	14.29	19.52	22.99	19.20	16.54	15.98	15.84	13.58	14.30	17.78	21.64	23.91	13.08	12.46	13.59	18.08				
XVIII (*n* = 6)	13.82	19.84	21.98	18.57	15.74	15.50	14.05	12.65	13.25	17.60	20.86	23.87	12.00	11.57	13.74	16.97	10.92			
XIX (*n* = 3)	18.26	21.96	22.35	20.02	17.40	09.59	17.79	17.92	14.47	18.97	22.40	24.93	18.81	18.03	21.03	18.40	18.84	18.27		
XX (*n* = 6)	13.30	16.82	19.14	16.17	13.28	12.97	09.14	11.89	09.69	14.91	18.85	21.43	12.54	12.53	16.15	13.52	13.17	12.13	16.18	
XXI (*n* = 10)	16.25	20.55	22.55	19.57	17.03	16.76	11.73	15.30	13.62	18.66	22.12	25.12	16.09	16.19	19.24	17.68	17.88	16.00	19.20	10.47

The number of base substitutions per site averaged across all sequence pairs between groups is shown. Analyses were conducted using the maximum composite likelihood model. The rate variation among sites was modeled with a gamma distribution (shape parameter = 1). The analysis involved 128 nucleotide sequences of the pilot dataset, including three DRC strains. The included codon positions were 1st, 2nd, 3rd, and noncoding. All positions containing gaps and missing data were eliminated. Evolutionary analyses were conducted in MEGA7 v 7.0.23. The number in brackets indicates the number of taxa in each group. DRC indicates the new variant NDV isolated from the DRC.

**Table 3:** The antigenic relatedness between the Congo isolates and vaccine strains.

**Virus**	**Antiserum to Virus ** ^**a**^			
	**PL33/18**	**PL219/19**	**I-2**	**B1**
NDV/Ck/DRC/PL33/2018	1024	512	256	512
NDV/Ck/DRC/PL219/2019	512	512	512	512
I-2 vaccine	512	512	512	512
B1 vaccine	256	1024	1024	512
**Virus**	**Antiserum to Virus ^b^**			
	**PL33/18**	**PL219/19**	**I-2**	**B1**
NDV/Ck/DRC/PL33/2018	1.00	1.00	0.98	0.99
NDV/Ck/DRC/PL219/2019	0.99	1.00	0.89	0.95
I-2 vaccine	0.98	0.98	1.00	0.97
B1 vaccine	0.90	1.00	0.99	1.00

^a^ The hemagglutination inhibition titer between homologous and heterologous viruses; the underline indicates the homologous titer. ^b^ The R-value was obtained from cross-serum neutralizing titers between the field and vaccine strains; bold indicates the homologous R-value.

## Data Availability

Not applicable.

## Supplementary Materials

The following are available online at https://www.mdpi.com/1999-4915/13/2/151/s1: Figure S1: The sampling location in Democratic Republic of the Congo (DRC). The blue shaded region indicates the sampling province in the DRC, Figure S2: Phylogenetic tree based on the whole-genome sequence of NDV class II. The tree was constructed in MEGA7 v 7.0.23 using the maximum-likelihood method based on a general time reversible model with 1000 bootstrap replicates. The dataset contains 64 taxa including three Congo strains which are indicated in red.
