# Supplementary material for: A New Variant among Newcastle Disease Viruses Isolated in the Democratic Republic of the Congo in 2018 and 2019
Source: Viruses. 2021 Jan 20;13(2):151. doi: 10.3390/v13020151 (PMC7909526; doi:10.3390/v13020151)
Supplement: Supplementary file 1 [file viruses-13-00151-s001.zip › viruses-1059102-supplementary.pptx]

## Slide 1
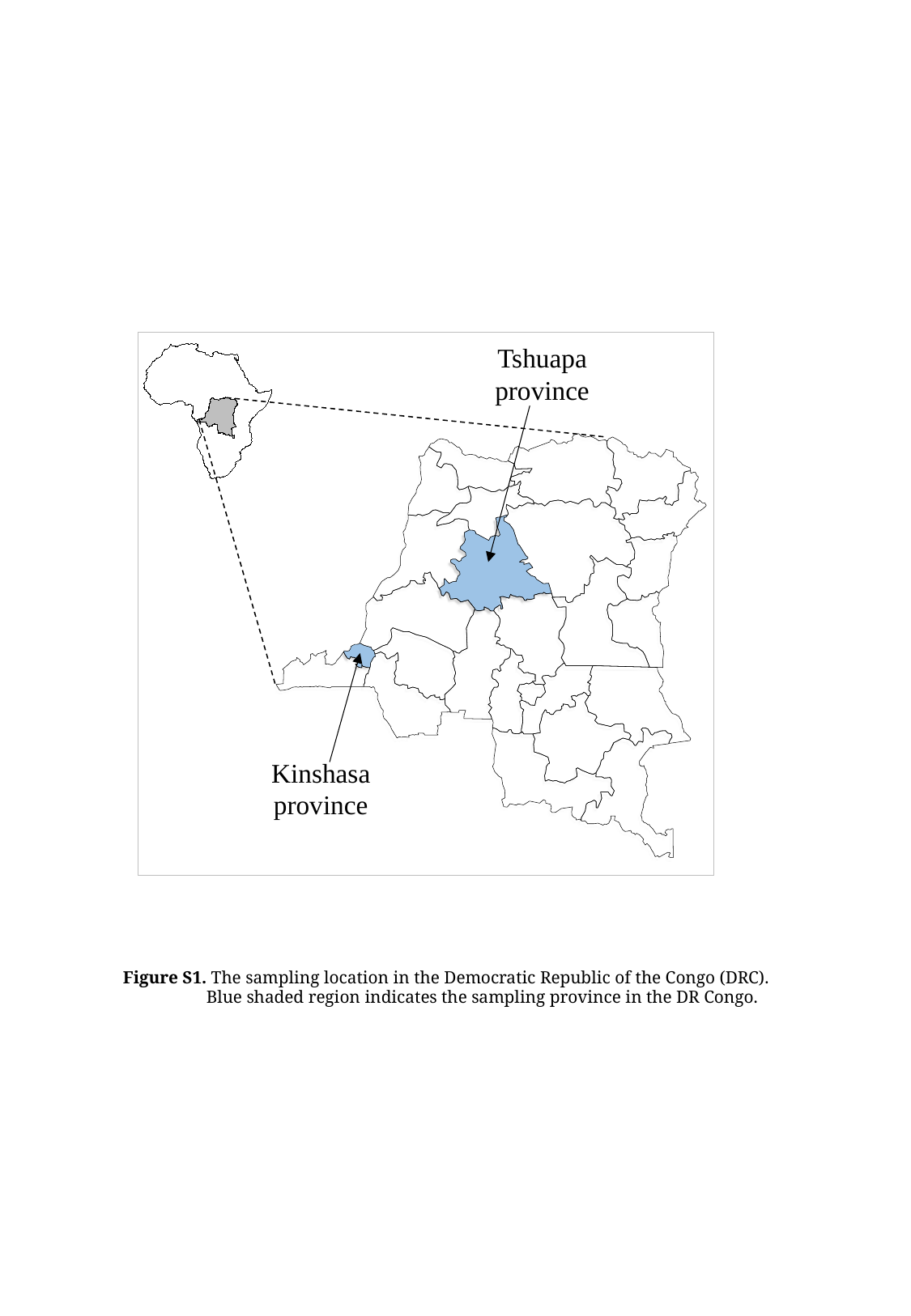

Tshuapa province
Kinshasa province
Figure S1. The sampling location in the Democratic Republic of the Congo (DRC). Blue shaded region indicates the sampling province in the DR Congo.

## Slide 2
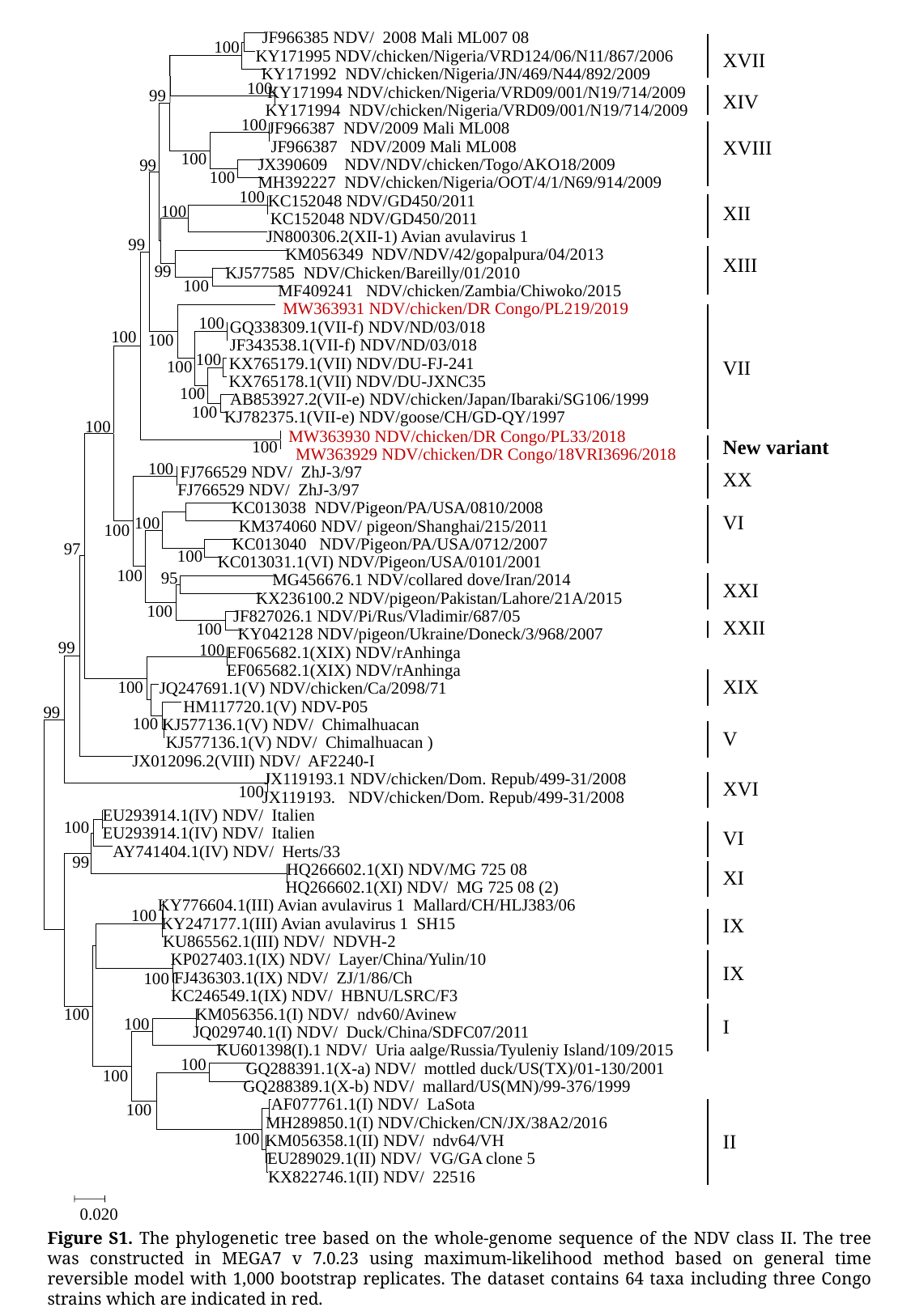

JF966385 NDV/ 2008 Mali ML007 08
 KY171995 NDV/chicken/Nigeria/VRD124/06/N11/867/2006
 KY171992 NDV/chicken/Nigeria/JN/469/N44/892/2009
 KY171994 NDV/chicken/Nigeria/VRD09/001/N19/714/2009
 KY171994 NDV/chicken/Nigeria/VRD09/001/N19/714/2009
 JF966387 NDV/2009 Mali ML008
 JF966387 NDV/2009 Mali ML008
 JX390609 NDV/NDV/chicken/Togo/AKO18/2009
 MH392227 NDV/chicken/Nigeria/OOT/4/1/N69/914/2009
 KC152048 NDV/GD450/2011
 KC152048 NDV/GD450/2011
 JN800306.2(XII-1) Avian avulavirus 1
 KM056349 NDV/NDV/42/gopalpura/04/2013
 KJ577585 NDV/Chicken/Bareilly/01/2010
 MF409241 NDV/chicken/Zambia/Chiwoko/2015
MW363931 NDV/chicken/DR Congo/PL219/2019
 GQ338309.1(VII-f) NDV/ND/03/018
 JF343538.1(VII-f) NDV/ND/03/018
 KX765179.1(VII) NDV/DU-FJ-241
 KX765178.1(VII) NDV/DU-JXNC35
 AB853927.2(VII-e) NDV/chicken/Japan/Ibaraki/SG106/1999
 KJ782375.1(VII-e) NDV/goose/CH/GD-QY/1997
 MW363930 NDV/chicken/DR Congo/PL33/2018
100
MW363929 NDV/chicken/DR Congo/18VRI3696/2018
 FJ766529 NDV/ ZhJ-3/97
 FJ766529 NDV/ ZhJ-3/97
 KC013038 NDV/Pigeon/PA/USA/0810/2008
 KM374060 NDV/ pigeon/Shanghai/215/2011
 KC013040 NDV/Pigeon/PA/USA/0712/2007
 KC013031.1(VI) NDV/Pigeon/USA/0101/2001
 MG456676.1 NDV/collared dove/Iran/2014
 KX236100.2 NDV/pigeon/Pakistan/Lahore/21A/2015
 JF827026.1 NDV/Pi/Rus/Vladimir/687/05
 KY042128 NDV/pigeon/Ukraine/Doneck/3/968/2007
 EF065682.1(XIX) NDV/rAnhinga
 EF065682.1(XIX) NDV/rAnhinga
 JQ247691.1(V) NDV/chicken/Ca/2098/71
 HM117720.1(V) NDV-P05
 KJ577136.1(V) NDV/ Chimalhuacan
 KJ577136.1(V) NDV/ Chimalhuacan )
 JX012096.2(VIII) NDV/ AF2240-I
 JX119193.1 NDV/chicken/Dom. Repub/499-31/2008
 JX119193. NDV/chicken/Dom. Repub/499-31/2008
 EU293914.1(IV) NDV/ Italien
 EU293914.1(IV) NDV/ Italien
 AY741404.1(IV) NDV/ Herts/33
 HQ266602.1(XI) NDV/MG 725 08
 HQ266602.1(XI) NDV/ MG 725 08 (2)
 KY776604.1(III) Avian avulavirus 1 Mallard/CH/HLJ383/06
100
 KY247177.1(III) Avian avulavirus 1 SH15
 KU865562.1(III) NDV/ NDVH-2
 KP027403.1(IX) NDV/ Layer/China/Yulin/10
 FJ436303.1(IX) NDV/ ZJ/1/86/Ch
 KC246549.1(IX) NDV/ HBNU/LSRC/F3
 KM056356.1(I) NDV/ ndv60/Avinew
100
 JQ029740.1(I) NDV/ Duck/China/SDFC07/2011
 KU601398(I).1 NDV/ Uria aalge/Russia/Tyuleniy Island/109/2015
 GQ288391.1(X-a) NDV/ mottled duck/US(TX)/01-130/2001
 GQ288389.1(X-b) NDV/ mallard/US(MN)/99-376/1999
 AF077761.1(I) NDV/ LaSota
 MH289850.1(I) NDV/Chicken/CN/JX/38A2/2016
100
 KM056358.1(II) NDV/ ndv64/VH
 EU289029.1(II) NDV/ VG/GA clone 5
 KX822746.1(II) NDV/ 22516
100
100
99
100
100
99
100
100
100
99
99
100
100
100
100
100
100
100
100
100
100
100
100
97
100
100
95
100
100
99
100
100
99
100
100
100
99
100
100
100
100
100
0.020
XVII
XIV
XVIII
XII
XIII
VII
New variant
XX
VI
XXI
XXII
XIX
V
XVI
VI
XI
IX
IX
I
II
Figure S1. The phylogenetic tree based on the whole-genome sequence of the NDV class II. The tree was constructed in MEGA7 v 7.0.23 using maximum-likelihood method based on general time reversible model with 1,000 bootstrap replicates. The dataset contains 64 taxa including three Congo strains which are indicated in red.
